# Spatial accessibility and inequality analysis of rabies-exposed patients to rabies post-exposure prophylaxis clinics in Guangzhou City, China

**DOI:** 10.1186/s12939-024-02207-2

**Published:** 2024-06-14

**Authors:** Jianguo Zhao, Min Luo, Xiaohua Tan, Zhihua Zhu, Meng Zhang, Jun Liu, Wenqing Lin, Yuwei Yang, Xing Li, Weilin Zeng, Dexin Gong, Zuhua Rong, Zitong Huang, Wenyuan Zheng, Huijie Guo, Siqing Zeng, Limei Sun, Jianpeng Xiao

**Affiliations:** 1 Guangdong Provincial Institute of Public Health, Guangdong Provincial Center for Disease Control and Prevention, Guangzhou, 511430 China; 2 Guangdong Provincial Center for Disease Control and Prevention, Guangzhou, 511430 China; 3 School of Public Health, Sun Yat-sen University, Guangzhou, 510080 China; 4 Department of Public Health and Preventive Medicine, School of Medicine, Ji’nan University, Guangzhou, 510632 China

**Keywords:** Rabies PEP clinics, Spatial accessibility, Inequality, 2SFCA method

## Abstract

**Background:**

The incidence of rabies exposure is high and increasing in China, leading to an urgent demand of rabies post-exposure prophylaxis (PEP) clinics for the injured. However, the spatial accessibility and inequality of rabies-exposed patients to rabies PEP clinics is less known in China.

**Methods:**

Based on rabies exposure data, PEP clinic data, and resident travel origin-destination (OD) matrix data in Guangzhou City, China, we first described the incidence of rabies exposure in Guangzhou from 2020 to 2022. Then, the Gaussian two-step floating catchment area method (2SFCA) was used to analyze the spatial accessibility of rabies-exposed patients to rabies PEP clinics in Guangzhou, and the *Gini* coefficient and Moran’s I statistics were utilized to evaluate the inequality and clustering of accessibility scores.

**Results:**

From 2020 to 2022, a total of 524,160 cases of rabies exposure were reported in Guangzhou, and the incidence showed a significant increasing trend, with an average annual incidence of 932.0/100,000. Spatial accessibility analysis revealed that the overall spatial accessibility scores for three scenarios (threshold of driving duration [d_0_] = 30 min, 45 min, and 60 min) were 0.30 (95% CI: 0.07, 0.87), 0.28 (95% CI: 0.11, 0.53) and 0.28 (95% CI: 0.14, 0.44), respectively. Conghua, Huangpu, Zengcheng and Nansha districts had the higher accessibility scores, while Haizhu, Liwan, and Yuexiu districts exhibited lower spatial accessibility scores. The *Gini* coefficient and Moran’s I statistics showed that there were certain inequality and clustering in the accessibility to rabies PEP clinics in Guangzhou.

**Conclusions:**

This study clarifies the heterogeneity of spatial accessibility to rabies PEP clinics, and provide valuable insights for resource allocation to achieve the WHO target of zero human dog-mediated rabies deaths by 2030.

**Supplementary Information:**

The online version contains supplementary material available at 10.1186/s12939-024-02207-2.

## Introduction

Rabies is a viral zoonotic disease caused by rabies virus, with a mortality rate of almost 100% [1]. According to the World Health Organization (WHO), rabies kills approximately 59,000 people every year and it is widespread in more than 150 countries, with 95% of cases in Africa and Asia [2]. In 2018, WHO and other partner organizations endorsed a target of zero human dog-mediated rabies deaths by 2030 (“Zero by 30”) [3]. In recent years, despite that the incidence of human rabies in China has shown a decreasing trend [4], the rabies exposure remains a major disease burden and exhibits a significant upward trend [5–8].

In many countries, rabies was successfully eliminated by vaccinating humans and dogs through the One Health approach [9]. However, the lack of strong political commitments and feasible plans in China made it difficult to vaccinate dogs, especially stray dogs [10]. Previous studies underscored the critical roles of timely and appropriately post exposure prophylaxis (PEP) in preventing rabies and the cost-effectiveness of improving accessibility to PEP clinics [11–13]. As the province with the highest population and largest economy in China, Guangdong province set up rabies PEP clinics in 2005 as special rabies post-exposure treatment sites throughout the province, providing wound treatment, vaccine and immunoglobulin vaccination services to improve the normalization of treatment of rabies exposure, which were also used as sentinel sites for monitoring the exposed population. In accordance with the *Guangdong Provincial Rabies Exposure Prevention and Disposal Clinic Management Guidelines (2020)*, clinics should be set up appropriately according to population density, service radius, geographic conditions and medical and healthcare resource allocation, and at least one PEP clinic should be set up in each subdistrict/town. However, few studies investigated the spatial accessibility of rabies-exposed patients to rabies PEP clinics. Spatial accessibility measures the accessibility or convenience of accessing facilities or services for residents, and is widely used in city planning, transportation logistics, and health service evaluation [14, 15]. Various methods exist for evaluating spatial accessibility, including shortest path analysis, Huff model, two-step floating catchment area method (2SFCA). Among them, the shortest path analysis method takes into account the distance factor, but does not take into account the scale of supply and demand points [16]. The Huff model considers facility scale and distance factors; however, the scale of demand points is not included in the model [17]. In contrast, the 2SFCA method is advantageous as it considers multiple factors such as distance, supply and demand point facilities, etc [18, 19]. This approach has been effectively employed in studies related to public health, such as assessing the spatial accessibility of COVID-19 patients to healthcare facilities in Florida [18], and evaluating the spatial accessibility of COVID-19 vaccine resources in the United States [19]. Therefore, in the context of the increasing incidence of rabies exposure, the 2SFCA method can more accurately assess the spatial accessibility of rabies exposed patients to rabies PEP clinics, which is of great significance for improving medical resource allocation and guiding future policy decisions.

As a mega city in China, Guangzhou, the capital of Guangdong has tens of thousands of people exposed to rabies who need vaccination per year, but the spatial accessibility and equality of rabies-exposed patients to rabies PEP clinics is less known. In this study, we chose Guangzhou as the study site and conducted a spatial accessibility analysis based on the 2SFCA method, while evaluating the fairness and clustering of accessibility scores. This study will clarify the accessibility of rabies-exposed patients to PEP clinics in Guangzhou, and help improve the efficiency of medical resource allocation, and provide study method references for other regions or similar studies.

## Materials and methods

### Study area and data sources

In this study, Guangzhou City was selected as the study area (Fig.S1). Guangzhou has over 18 million inhabitants and a high incidence of rabies exposure every year [8]. It consists of 11 districts, namely Yuexiu, Liwan, Haizhu, Tianhe, Baiyun, Huangpu, Panyu, Huadu, Nansha, Zengcheng, and Conghua. This study was based on multi-source data, including rabies exposure data, PEP clinic information, population data, administrative map data, and resident travel origin-destination(OD) matrix data in Guangzhou. Rabies exposure is defined as patients who have been bitten, scratched, licked mucous membranes or damaged skin by rabies, suspected rabies, or host animals that cannot be determined whether they have rabies [1].

The data of rabies exposure ranging from 2020 to 2022 and the rabies PEP clinic data were obtained from Guangdong Provincial Center for Disease Control and Prevention (GDCDC). The rabies exposure data included various information, such as the address and exposure levels (I, II, III) of people exposed to rabies.

The PEP clinic information records detailed information including the name, institution code, and address in Guangzhou, which were obtained from the Guangdong Provincial Center for Disease Control and Prevention.

The population data from 2020 to 2022 were obtained from the Guangzhou Statistical Yearbook [20], which were used to calculate the annual incidence of rabies exposure. The population data for each subdistrict/town in Guangzhou was collected from the seventh population census data, which contains the total population of 176 subdistricts/towns of Guangzhou in 2020 [21].

The administrative map data was obtain from Resource and Environment Science and Data Center (https://www.resdc.cn/Default.aspx).

The resident travel OD matrix data was obtained using the Direction API of Baidu Maps, one of the largest map service operators in China (https://lbsyun.baidu.com). The Direction API supports the planning for driving, cycling, walking, and public transportation routes in Mainland China, providing accurate travel paths and times from the origin to the destination [22]. In this study, we converted the address data of residential (administrative district center of each subdistrict/town) and PEP clinics into geographical coordinates under the WGS84 standard, and then obtained the OD matrix data of driving between them through the Direction API.

### Methods

#### Descriptive analysis

We calculated the annual average incidence of rabies exposure based on the data of rabies exposure and population from 2020 to 2022. Trend test was employed to examine the trend of the rabies exposure incidence.

#### Spatial accessibility analysis

According to the Technical Guidelines for Human Rabies (2016) released by the China Provincial Center for Disease Control and Prevention [1], people with WHO category II exposure or category III exposure should immediately accept PEP (Category II: vaccine, Category III: vaccine and Rabies Immune Globulin) as an emergency procedure. Thus, we used the average exposed population with WHO category II exposure and category III exposure of each subdistrict/town from 2020 to 2022 as the demand population(${D}_{i}$).

The two-step floating catchment area(2SFCA) method is a widely used approach for evaluating the accessibility of facilities or services [18, 19, 23]. Compared to other methods (e.g. shortest path analysis, Huff model), it takes into account the scale of both supply and demand points for analysis, aligning more closely with the real-world scenarios [16, 24]. In this study, considering to the limitations in the service radius of rabies PEP clinics, we used Gaussian 2SFCA method to analyze the spatial accessibility of rabies-exposed patients to rabies PEP clinics [25–27]. The first step of this method was to generate a catchment area with a threshold (d_0_) centered on the rabies PEP clinic ${S}_{j}$. Within this catchment area, all potential demanders were calculated, where each demander${D}_{i}$ was weighted using a Gaussian equation (Eq. 1). We then computed the ratio ${R}_{j}$of each clinic based on its potential demand (Eq. 2).

1 $$G\left({d}_{ij},{d}_{0}\right)=\left\{\begin{array}{c}\frac{{e}^{-\frac{1}{2}\cdot \big(\frac{{d}_{ij}}{{d}_{0}}\big)^2}-{e}^{-\frac{1}{2}}}{1-{e}^{-\frac{1}{2}}}, \; {d}_{ij}\leq{d}_{0}\\  \quad\quad 0,\quad\quad\quad\quad {d}_{ij}>{d}_{0} \end{array}\right.$$

2 $${R}_{j}=\frac{{S}_{j}}{\sum _{i\in ({d}_{ij}\le {d}_{0})}G({d}_{ij},{d}_{0})\cdot {D}_{i}}$$

where ${d}_{ij}$ is the travel distance between each clinic and the subdistrict/town. Referring to the plan in Anhui and Shaanxi provinces, China [28, 29], where the standard driving time for patients with rabies exposure to the clinic was less than 1 h, we set the three threshold scenarios (i.e. Short-distance driving scenario 1, d_0_ = 30 min; Medium-distance driving scenario 2, d_0_ = 45 min; Long-distance driving scenario 3, d_0_ = 60 min) to evaluate the accessibility of patients to rabies PEP clinics.

The second step was to search clinics in each demand location within a threshold of d_0_, and then sum the Gaussian weighted ratio for each clinic to obtain the spatial accessibility score ${A}_{i}$ of each demand location (Eq. 3). The ${A}_{i}$ essentially represents the supply-demand ratio between outpatient clinics and patients. In this study, it represents the number of PEP clinics that each person exposed to rabies in different subdistricts/towns can obtain.

3 $${A}_{i}={\sum }_{i\in ({d}_{ij}\le {d}_{0})}G\left({d}_{ij},{d}_{0}\right)\cdot {R}_{j}$$

#### Inequality and clustering analysis

We further conducted an inequality analysis on accessibility scores based on *Gini* coefficient and Lorentz curve. And the *Gini* coefficient was given as [30]:

4 $$Gini = \frac{{\sum }_{i\in n}\sum _{j\in n}\left|{x}_{i}-{x}_{j}\right|}{2{n}^{2}\stackrel{-}{x}}$$

Where ${x}_{i}$and ${x}_{j}$ denote the accessibility scores for the i-th and j-th subdistricts, respectively, *n* represent the number of subdistricts. When the *Gini* coefficient is below 0.2, it means that the allocation of medical resources is absolutely fair; 0.2 to 0.3 means that resources are relatively average with slight gaps; 0.3 to 0.4 means resources are relatively reasonable with a certain gap; 0.4 to 0.5 indicates a large gap in resource allocation; greater than 0.5 indicates a huge gap in resource allocation [31]. And we conducted a spatial clustering analysis on accessibility scores under three scenarios based on Moran’s index result from global and local spatial autocorrelation analysis [32].

#### Sensitivity analysis

A previous study showed that the COVID-19 intervention may impact the epidemic of rabies during the COVID-19 Pandemic in 2020 [33]. To account for this impact and ensure the robustness of our findings, we employed the data of rabies exposure during 2021–2022 (excluding 2020) in our model for sensitivity analysis and compared the difference in the results.

All the above statistical analyses were conducted with R software (version 4.0.3, R Foundation for Statistical Computing, Vienna, Austria). The “dplyr”, “RCurl” and “XML” packages were used to process data and web page information; “trend”, “spdep” and “lctools”packages were used for statistical analysis; all figures were generated with “ggplot2” package. All statistical tests were two-sided, and *P* < 0.05 was considered statistically significant.

## Results

### General characteristics

**Fig. 1:**
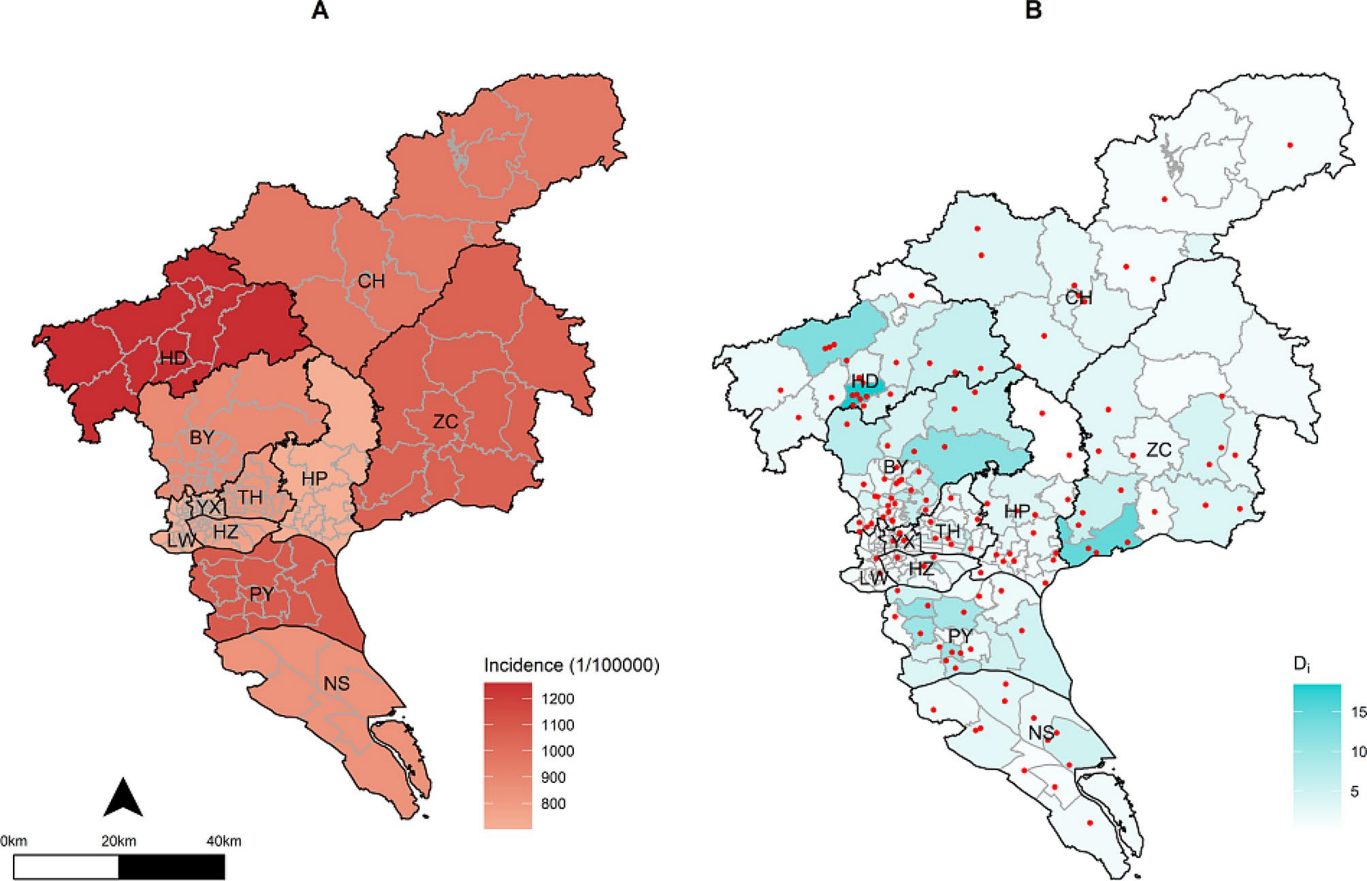
The incidence of rabies exposure and clinic distribution. **(A)** The total incidence of Category II and Category III exposure by district during 2020–2022. **(B)** Clinic distribution (red dots) and the average number of people exposed to rabies (${D}_{i}$) per day during 2020–2022. *Abbreviations* LW = Liwan; YX = Yuexiu; HZ = Haizhu; TH = Tianhe; BY = Baiyun; HP = Huangpu; PY = Panyu; HD = Huadu; NS = Nansha; ZC = Zengcheng; CH = Conghua

From 2020 to 2022, the reported number of potential rabies exposed in Guangzhou was 524,160, and the annual average incidence was 932.0/100,000. The highest incidence rate was in 2022, reaching 1,167.0/100,000, of which category II and III exposure cases accounted for 1,123.4/100,000 (96.3%). In contrast, the lowest incidence was observed in 2020 at 618.7/100,000. The total incidence of rabies exposure showed a significant increasing trend annually (*P* < 0.05)(Table 1). For different districts, the average annual incidence of each district in 2020–2022 was between 702.5/100,000 and 1,259.4/100,000. Among them, three districts with the highest incidence were Huadu District, Panyu District and Zengcheng District, while the lowest incidence were recorded in Liwan District, Huangpu District and Yuexiu District (Fig. 1.A and Table S1).

**Table 1:** Epidemiological characteristics of human rabies exposure in Guangzhou from 2020 to 2022

Years	Total cases	Category I exposure(%)	Category II exposure(%)	Category III exposure (%)	Unclassified (%)
2020	115,941	4,684(4.04)	83,245(71.80)	27,370(23.61)	642(0.55)
2021	189,519	6,209(3.28)	121,863(64.30)	60,523(31.94)	924(0.50)
2022	218,700	7,301(3.34)	133,210(60.91)	77,318(35.35)	871(0.40)
Mean	174,720	6,065(3.47)	112,773(64.54)	55,070(31.52)	812(0.46)

There were a total 137 rabies PEP clinics in Guangzhou, with an average of 0.73 clinics per 100,000 people. Among them, Conghua District had the highest per capita number of clinics with 1.53 per 100,000 people, while Liwan and Haizhu District had the lowest with 0.16 per 100,000 people(Table S1). Figure 1.B displayed that the spatial distribution of rabies PEP clinics were mainly concentrated in the central urban areas of Guangzhou (e.g., Yuexiu, Liwan, Haizhu, the southern part of Baiyun, Tianhe, etc.). These areas serve as the political and economic hubs of Guangzhou and exhibit relatively high population density. In contrast, medical clinics in Conghua, Zengcheng, Nansha, and other districts were more scattered. The number of exposed individuals were mainly concentrated in Huadu District(e.g., Xinhua subdistrict and Shiling town), Zengcheng District (e.g., Xintang town and Yongning subdistrict), and Panyu District(e.g., Dashi subdistrict and Shiqiao subdistrict).

### Spatial accessibility analysis

**Fig. 2:**
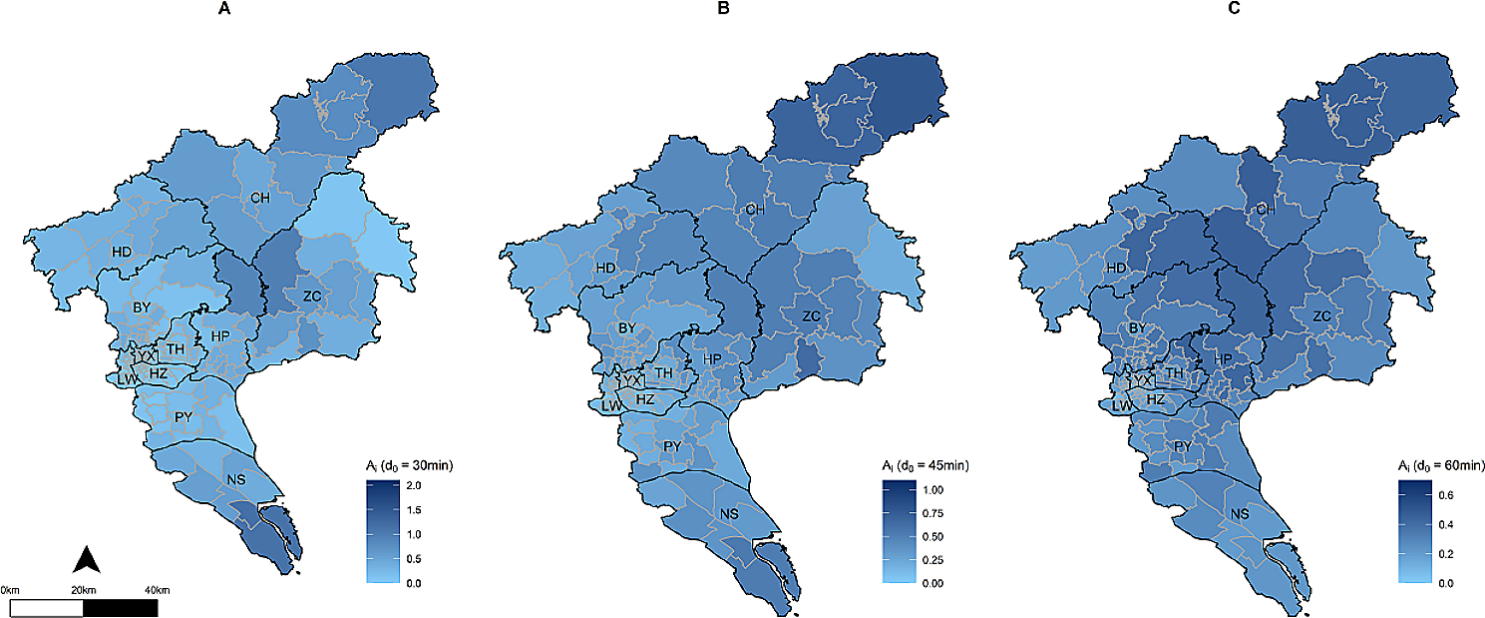
Accessibility analysis of rabies-exposed patients to rabies PEP clinics at different scenarios in Guangzhou. **(A)** Spatial accessibility score of each subdistrict/town under scenario1, d_0_ = 30 min; **(B)** Spatial accessibility score of each subdistrict/town under scenario 2, d_0_ = 45 min; **(C)** Spatial accessibility score of each subdistrict/town under scenario 3, d_0_ = 60 min

The spatial accessibility of rabies-exposed patients to rabies PEP clinics under three different scenarios was estimated (Fig. 2). At the city level, the overall spatial accessibility scores under three scenarios (d_0_ = 30 min, 45 min, and 60 min) were 0.30 (95% CI: 0.07, 0.87), 0.28 (95% CI: 0.11, 0.53) and 0.28 (95% CI: 0.14, 0.44), respectively. Notably, there were significant differences of spatial accessibility scores across districts. Under the three scenarios, the results showed that Conghua, Huangpu, Zengcheng and Nansha districts had the highest accessibility, while Haizhu, Liwan, and Yuexiu districts recorded the lowest spatial accessibility score (Table S2). At the subdistrict/town scale, differences in accessibility scores exist even among those located in the same district. For example, in the scenario of d_0_ = 30 min, the accessibility of Zhujiang subdistrict of Nansha District was 1.17, while the Longxue subdistrict in the same District, revealed a score of 0.28(Table S3).

### Inequality and spatial heterogeneity analysis

**Fig. 3:**
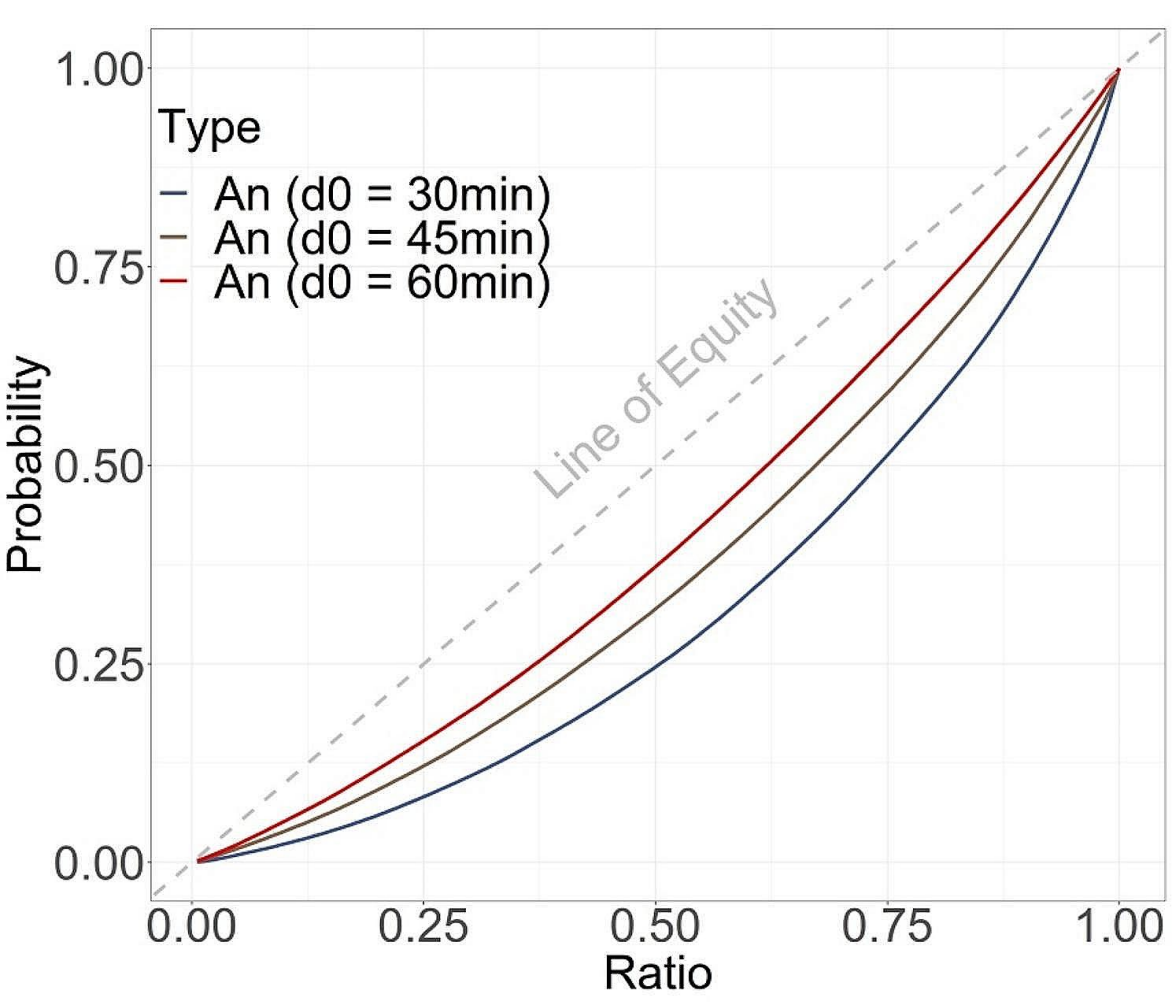
Lorenz curve based on the accessibility of exposed individuals at the subdistrict level in Guangzhou

Under three different scenarios, we found that as the driving time decreased, the inequality of accessibility significantly increased, manifested as a larger area between completely equal line and Lorentz curves (Fig. 3). The *Gini* coefficient under the three thresholds were *Gini*_*30min*_ *=* 0.36, *Gini*_*45min*_ *=* 0.25 and *Gini*_*60min*_ = 0.18, respectively.

**Fig. 4:**
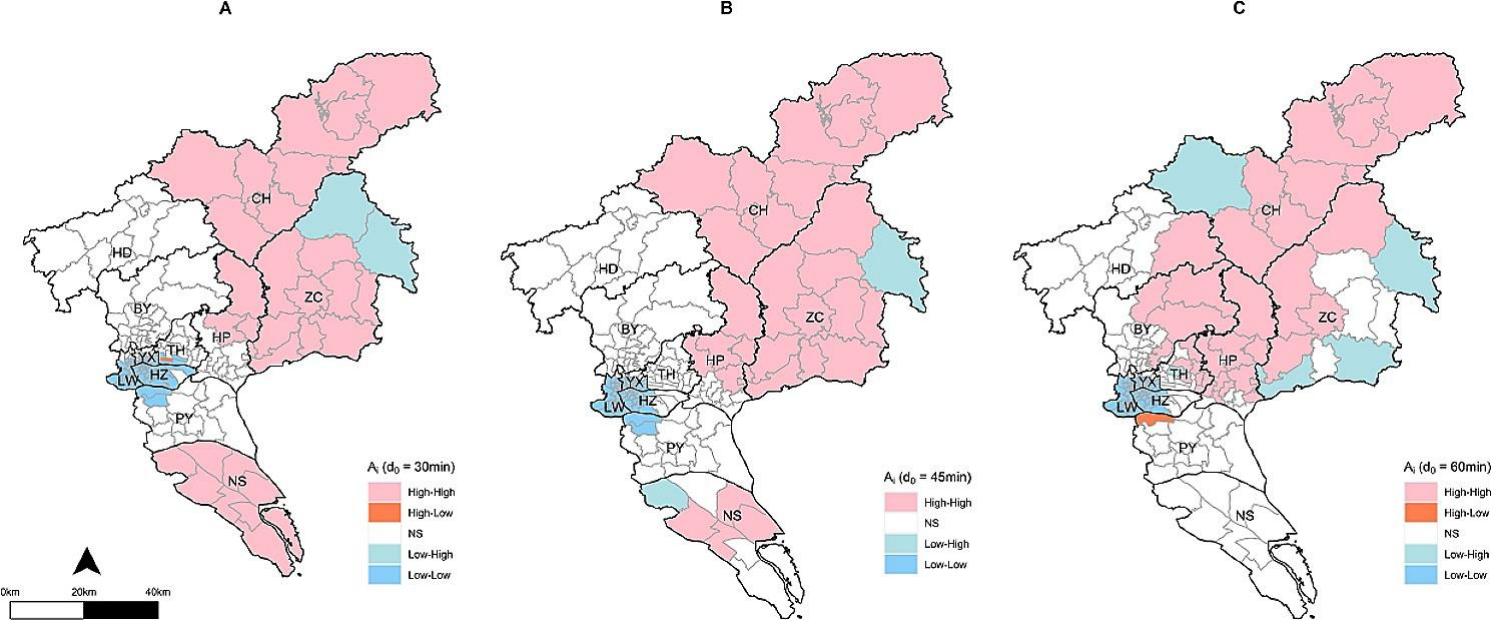
Cluster analysis under three scenarios with Anselin Moran’s I analysis. **(A)** Scenario 1: d_0_ = 30 min; **(B)** Scenario 2, d_0_ = 45 min; **(C)** Scenario 3, d_0_ = 60 min. (NS: Not Significant)

Table S4 displayed a significant positive spatial autocorrelation in spatial accessibility scores under three scenarios, and the corresponding global Moran’s I ranged from 0.598 to 0.708 (*P* < 0.05). Figure 4 further illustrated the local spatial clustering based on Anselin Moran’s I. The results showed that under the three scenarios, the accessibility score of Yuexiu, Liwan, Haizhu, and the southwest of Tianhe showed low value clustering (Low-Low), while high value clustering (High-High) mainly concentrated in the northeast and southern of Guangzhou, including Huangpu District, Conghua District, Zengcheng District, and Nansha District (excluding Scenario 3). These results demonstrated that there was spatial heterogeneity in the accessibility scores of rabies-exposed patients to rabies PEP clinics in Guangzhou, and the scores were lower in the central urban area.

### Sensitivity analysis

Based on the data of rabies exposure during 2021–2022 (excluding 2020), the sensitivity analysis (Table S5) showed that the overall spatial accessibility scores under different scenarios were 0.25, 0.24 and 0.24, respectively. These scores were close to the results based on the data of rabies exposure during 2020–2022, with no significant differences observed (*P* > 0.05).

## Discussion

Rabies remains a significant public health issue that cannot be ignored, and post-exposure prophylaxis is one of the best approaches to prevent rabies. In this study, we found that the incidence of rabies exposure in Guangzhou showed an upward trend from 2020 to 2022, which was consistent with previous studies [34]. The annual incidence of rabies exposure in Guangzhou City was 932.0/100,000, similar to that in Zhengzhou City (960.1/100,000)and Tianjin City (909.66/100,000) in China [7, 35]. At the district level, Huadu, Zengcheng and Panyu districts had the highest incidence of rabies exposure. The reason may be that these areas have a higher proportion of rural areas, and most dogs are free-roaming, which indirectly increases the risk of rabies exposure [8, 36].

In our study, the overall spatial accessibility scores under three scenarios were 0.30, 0.28 and 0.28, respectively. This means each PEP clinic was needed to serve 3.30 (= 1/0.30) to 3.57 (= 1/0.28) people exposed to rabies per day in Guangzhou, which was higher than other provinces, such as Zhejiang province (= 2.72) and Hunan province (= 0.85) in China. It may be related to the lower number of outpatient clinics per capita in Guangzhou [37]. Given the increasing number of individuals exposed to rabies, it is necessary to increase the number of PEP clinics or improve vaccination services to adapt to this trend and better serve patients.

We found that the *Gini* coefficient was 0.36 under the scenario of d_0_ = 30 min, showing inequality among subdistricts/towns, and the spatial accessibility in Yuexiu, Liwan, Haizhu, and the southwest of Tianhe was lower than that in other districts and showed a Low-Low clustering. A previous study showed that the distribution of rabies exposure in Guangzhou was shifting from rural to these areas, with an increasing risk of exposure caused by pet dogs or other animals [8]. And the population density in these districts is over ten times higher than that of other districts, indirectly increasing the demand for PEP clinics (Fig.S1). On the other hand, congested traffic will also reduce the spatial accessibility by increasing the time to reach the PEP clinics. Monitoring data revealed that these areas of Guangzhou were almost in a moderate congestion state [38]. In future planning, the government and relevant departments in urban areas can increase the number of rabies PEP clinics to meet the huge demand, while allocating resources reasonably based on the demand differences of the districts.

The results also indicated that the spatial accessibility of the northeast and southeast of Guangzhou was higher than that of the central urban area. However, it is important to note that the risk of rabies is not only related to the accessibility to rabies PEP clinics, but also on the management of dogs and people’s awareness of preventive measures after being exposed to rabies [5, 39]. Geographically, these places are far from the center of Guangzhou with a high proportion of rural areas. A previous study showed most dogs in rural areas roamed freely and were not required registration, posing a significant risk of infection and transmission of rabies [5]. In addition, people in rural areas had weak awareness of rabies prevention [40–42].Previous studies showed rural areas in China were still the main source of rabies transmission, and improving the awareness of rabies prevention in rural areas is still one of the main measures to eliminate human rabies [8, 43, 44].

The Gaussian 2SFCA method combined with real-time route planning from Baidu Maps in this study was the first time in China to evaluate the accessibility of patients to rabies PEP clinics, which can more accurately evaluate the accessibility to outpatient services. Previous studies showed that the travel time/distance can affect the accessibility scores to service facilities [45–47]. In this study, we used Baidu Maps to obtain patient’s travel time, which was a more realistic approach than calculations based on Euclidean distance or road standard speed. The shortest travel time calculated by Euclidean distance ignores the design of the road and underestimates the time it takes for demanders to reach the service facilities [46]. On the other hand, the time obtained based on road network and standard speed ignores various factors that affect travel, such as traffic congestion and traffic lights, etc [16, 45]. In contrast, Baidu Maps’ route planning takes into consideration factors like traffic flow, road restrictions, and other variables that better reflect real-life situations, which can also be employed in similar studies.

Some limitations should be mentioned. First, our study only focused on the accessibility to rabies PEP clinics, without analyzing the economic burden of canine vaccination, which may restrict residents from getting vaccination [5, 48, 49]. Moreover, the spatial accessibility scores are also influenced by the capacity of rabies PEP clinics, such as the number of medical staff and vaccine supply. However, the lack of comprehensive indicators for assessing outpatient service capacity restricts a more detailed evaluation. Thirdly, the administrative center of each subdistrict/town was used as the residential address, rather than using location points of rabies-exposed patients for analysis, which may also affect the accuracy of spatial accessibility. Finally, this study is based on the rabies exposure data from 2020 to 2022. During this period, the world is experiencing the COVID-19 pandemic, and China has taken strict nonpharmaceutical interventions (NPIs) against COVID-19. Stringent NPIs not only reduced the incidence of COVID-19, but also affected the incidence of other infectious diseases, including rabies, which may bias our accessibility scores [33]. However, based on the sensitivity analysis, we found that these differences were not statistically significant.

## Conclusion

In conclusion, this is the first study to explore the spatial accessibility and inequality of rabies-exposed patients to rabies PEP clinics at subdistrict/town scale in China, and the method used in this study can be extended to other similar studies. The incidence of rabies exposure showed a significant upward trend in Guangzhou from 2020 to 2022. Notably, the spatial accessibility to PEP clinics is lower in the urban region, and exhibiting a certain inequality. These results can help to clarify the heterogeneity of spatial accessibility, and provide valuable insights for resource allocation to achieve the WHO target of “Zero by 30”.

### Electronic supplementary material

Below is the link to the electronic supplementary material.

Supplementary Material 1

## Data Availability

The rabies PEP clinic information, population data, administrative map data and resident travel OD matrix data in this study are available in the Guangdong Provincial Center for Disease Control and Prevention - GDCDC (http://cdcp.gd.gov.cn), Guangzhou Statistics Bureau(http://tjj.gz.gov.cn/), Resource and Environment Science and Data Center(https://www.resdc.cn/Default.aspx) and Baidu maps (https://lbsyun.baidu.com/products/direction), respectively. The data of rabies exposure is not publicly available due to the non-disclosure agreements but are available from the corresponding author on reasonable request.

## Abbreviations

PEP: Post-exposure prophylaxis
OD matrix: Origin-Destination matrix
2SFCA: Two-step floating catchment area
WHO: World Health Organization
GDCDC: Guangdong Provincial Center for Disease Control and Prevention
COVID-19: Corona virus disease 2019
