# Supplementary material for: Spatial accessibility and inequality analysis of rabies-exposed patients to rabies post-exposure prophylaxis clinics in Guangzhou City, China
Source: Int J Equity Health. 2024 Jun 14;23:122. doi: 10.1186/s12939-024-02207-2 (PMC11179278; doi:10.1186/s12939-024-02207-2)
Supplement: Supplementary file 1 — Supplementary Material 1 [file 12939_2024_2207_MOESM1_ESM.docx]

**Supplemental Materials**

**Fig. S1** The population density (Pop density) of different districts in Guangzhou, the capital of Guangdong, China. Abbreviations: LW = Liwan; YX = Yuexiu; HZ = Haizhu; TH = Tianhe; BY = Baiyun; HP = Huangpu; PY = Panyu; HD = Huadu; NS = Nansha; ZC = Zengcheng; CH = Conghua.

**Table S1** Average incidence and rabies PEP clinic in 11 districts of Guangzhou during 2020-2022.

**Table S2** Average spatial accessibility $A_{i}$(95%CI) of different districts in Guangzhou under different thresholds.

**Table S3** Spatial accessibility scores $A_{i}$ of different subdistricts/Towns in Guangzhou under different thresholds.

**Table S4**  Spatial autocorrelation statistics under different thresholds.

**Table S5** Sensitivity analyses of accessibility scores using different data of rabies exposure (during 2020-2022 *vs* during 2021-2022).


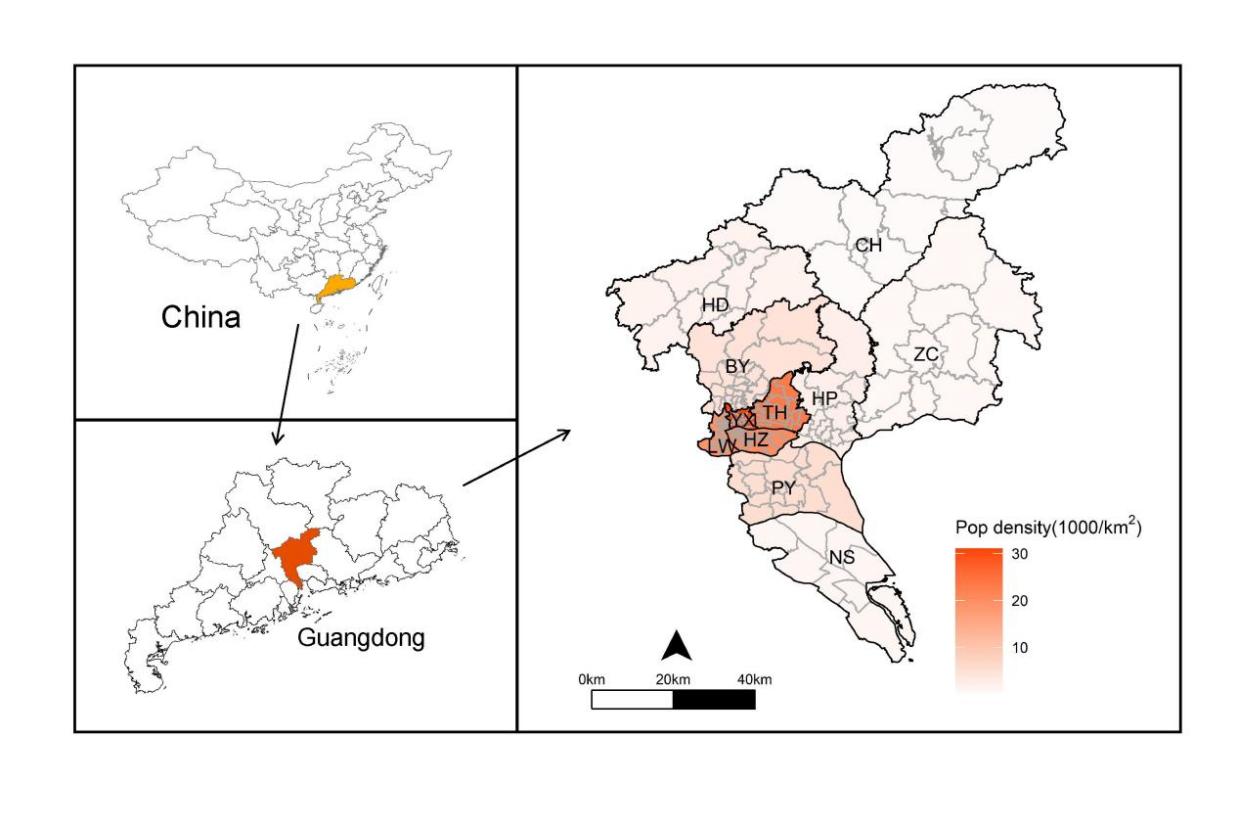


**Fig. S1** The population density (Pop density) of different districts in Guangzhou, the capital of Guangdong, China.Abbreviations: LW = Liwan; YX = Yuexiu; HZ = Haizhu; TH = Tianhe; BY = Baiyun; HP = Huangpu; PY = Panyu; HD = Huadu; NS = Nansha; ZC = Zengcheng; CH = Conghua.

**Table S1** Average incidence and rabies PEP clinic in 11 districts of Guangzhou during 2020-2022.

| District | Average number of people exposed to rabies* | Incidence(/100,000) | Number of PEP clinics | Number of PEP clinics per capita |
| --- | --- | --- | --- | --- |
| Huadu | 20684 | 1259.4 | 21 | 1.28 |
| Panyu | 29039 | 1092.4 | 14 | 0.53 |
| Zengcheng | 15546 | 1060.2 | 18 | 1.23 |
| Conghua | 6700 | 965.6 | 11 | 1.53 |
| Baiyun | 32989 | 881.4 | 29 | 0.77 |
| Nansha | 7156 | 845.2 | 12 | 1.42 |
| Tianhe | 18573 | 828.5 | 7 | 0.31 |
| Haizhu | 14022 | 770.8 | 3 | 0.16 |
| Liwan | 8907 | 719.3 | 2 | 0.16 |
| Huangpu | 10128 | 718.5 | 16 | 1.27 |
| Yuexiu | 7296 | 702.5 | 4 | 0.39 |

*：excluding people exposed to rabies with miss address.

**Table S2** Average spatial accessibility $A_{i}$(95%CI) of different districts in Guangzhou under different thresholds.

| District | $A_{i}$(d0=30 min) | $A_{i}$(d0=45 min) | $A_{i}$(d0=60 min) |
| --- | --- | --- | --- |
| Conghua | 0.66(0.48~1.03) | 0.56(0.45~0.78) | 0.40(0.29~0.47) |
| Nansha | 0.61(0.29~1.17) | 0.39(0.28~0.55) | 0.24(0.15~0.31) |
| Zengcheng | 0.51(0.09~0.91) | 0.46(0.24~0.62) | 0.34(0.24~0.42) |
| Huangpu | 0.46(0.24~0.84) | 0.35(0.26~0.48) | 0.35(0.27~0.44) |
| Huadu | 0.36(0.25~0.51) | 0.32(0.21~0.45) | 0.29(0.20~0.43) |
| Baiyun | 0.30(0.14~0.41) | 0.30(0.19~0.37) | 0.33(0.24~0.39) |
| Panyu | 0.23(0.12~0.33) | 0.27(0.17~0.38) | 0.29(0.21~0.34) |
| Tianhe | 0.22(0.11~0.32) | 0.28(0.17~0.42) | 0.33(0.22~0.45) |
| Yuexiu | 0.17(0.08~0.30) | 0.17(0.12~0.24) | 0.20(0.15~0.27) |
| Liwan | 0.14(0.03~0.28) | 0.15(0.07~0.23) | 0.19(0.10~0.25) |
| Haizhu | 0.10(0.07~0.15) | 0.15(0.11~0.23) | 0.19(0.13~0.30) |

**Table S3** Spatial accessibility scores $A_{i}$ of different subdistricts/Towns in Guangzhou under different thresholds.

| Subdistrict/Town | District | $A_{i}$(d0=30 min) | $A_{i}$(d0=45 min) | $A_{i}$(d0=60 min) |
| --- | --- | --- | --- | --- |
| Shamian subdistrict | Liwan | 0.03 | 0.09 | 0.11 |
| Lingnan subdistrict | Liwan | 0.24 | 0.21 | 0.24 |
| Hualin subdistrict | Liwan | 0.04 | 0.09 | 0.13 |
| Duobao subdistrict | Liwan | 0.08 | 0.12 | 0.15 |
| Changhua subdistrict | Liwan | 0.09 | 0.12 | 0.16 |
| Fengyuan subdistrict | Liwan | 0.19 | 0.17 | 0.20 |
| Longjun subdistrict | Liwan | 0.17 | 0.16 | 0.19 |
| Jinhua subdistrict | Liwan | 0.21 | 0.18 | 0.21 |
| Caihong subdistrict | Liwan | 0.23 | 0.20 | 0.23 |
| Nanyuan subdistrict | Liwan | 0.33 | 0.24 | 0.25 |
| Xicun Subdistrict | Liwan | 0.22 | 0.19 | 0.21 |
| Zhanqian Subdistrict | Liwan | 0.20 | 0.19 | 0.22 |
| Qiaozhong Subdistrict | Liwan | 0.23 | 0.21 | 0.24 |
| Baihedong Subdistrict | Liwan | 0.08 | 0.12 | 0.16 |
| Chongkou Subdistrict | Liwan | 0.11 | 0.13 | 0.18 |
| Huadi Subdistrict | Liwan | 0.20 | 0.20 | 0.25 |
| Shiweitang Subdistrict | Liwan | 0.15 | 0.16 | 0.19 |
| Chajiao Subdistrict | Liwan | 0.03 | 0.06 | 0.09 |
| Dongjiao Subdistrict | Liwan | 0.07 | 0.11 | 0.17 |
| Hailong Subdistrict | Liwan | 0.09 | 0.13 | 0.19 |
| Dongsha Subdistrict | Liwan | 0.07 | 0.11 | 0.16 |
| Zhongnan Subdistrict | Liwan | 0.11 | 0.14 | 0.20 |
| Hongqiao Subdistrict | Yuexiu | 0.31 | 0.24 | 0.26 |
| Beijing Subdistrict | Yuexiu | 0.18 | 0.17 | 0.19 |
| Liurong Subdistrict | Yuexiu | 0.28 | 0.21 | 0.23 |
| Liuhua Subdistrict | Yuexiu | 0.25 | 0.24 | 0.28 |
| Guangta Subdistrict | Yuexiu | 0.15 | 0.14 | 0.17 |
| Renmin Subdistrict | Yuexiu | 0.08 | 0.11 | 0.14 |
| Dongshan Subdistrict | Yuexiu | 0.08 | 0.13 | 0.16 |
| Nonglin Subdistrict | Yuexiu | 0.11 | 0.14 | 0.17 |
| Meihuacun Subdistrict | Yuexiu | 0.12 | 0.16 | 0.20 |
| Huanghuagang Subdistrict | Yuexiu | 0.14 | 0.15 | 0.21 |
| Huale Subdistrict | Yuexiu | 0.16 | 0.17 | 0.20 |
| Jianshe Subdistrict | Yuexiu | 0.16 | 0.17 | 0.20 |
| Datang Subdistrict | Yuexiu | 0.20 | 0.18 | 0.21 |
| Zhuguang Subdistrict | Yuexiu | 0.09 | 0.12 | 0.16 |
| Dadong Subdistrict | Yuexiu | 0.14 | 0.16 | 0.20 |
| Baiyun Subdistrict | Yuexiu | 0.20 | 0.19 | 0.22 |
| Dengfeng Subdistrict | Yuexiu | 0.26 | 0.24 | 0.26 |
| Kuangquan Subdistrict | Yuexiu | 0.20 | 0.17 | 0.21 |
| Chigang Subdistrict | Haizhu | 0.08 | 0.12 | 0.17 |
| Xingang Subdistrict | Haizhu | 0.08 | 0.11 | 0.14 |
| Changgang Subdistrict | Haizhu | 0.10 | 0.13 | 0.16 |
| Jiangnanzhong Subdistrict | Haizhu | 0.08 | 0.11 | 0.13 |
| Binjiang Subdistrict | Haizhu | 0.10 | 0.13 | 0.17 |
| Sushe Subdistrict | Haizhu | 0.10 | 0.13 | 0.16 |
| Hoitong Subdistrict | Haizhu | 0.07 | 0.11 | 0.13 |
| Nanhua West Subdistrict | Haizhu | 0.11 | 0.13 | 0.17 |
| Longfeng Subdistrict | Haizhu | 0.16 | 0.16 | 0.19 |
| Shayuan Subdistrict | Haizhu | 0.12 | 0.15 | 0.17 |
| Nanshitou Subdistrict | Haizhu | 0.14 | 0.16 | 0.20 |
| Fengyang Subdistrict | Haizhu | 0.08 | 0.12 | 0.16 |
| Ruibao Subdistrict | Haizhu | 0.11 | 0.17 | 0.20 |
| Jianghai Subdistrict | Haizhu | 0.06 | 0.11 | 0.17 |
| Pazhou Subdistrict | Haizhu | 0.14 | 0.24 | 0.33 |
| Nanzhou Subdistrict | Haizhu | 0.10 | 0.16 | 0.19 |
| Huazhou Subdistrict | Haizhu | 0.13 | 0.21 | 0.26 |
| Guanzhou Subdistrict | Haizhu | 0.15 | 0.21 | 0.27 |
| Wushan Subdistrict | Tianhe | 0.09 | 0.14 | 0.21 |
| Yuancun Subdistrict | Tianhe | 0.13 | 0.26 | 0.34 |
| Chebei Subdistrict | Tianhe | 0.23 | 0.25 | 0.31 |
| Shahe Subdistrict | Tianhe | 0.19 | 0.22 | 0.25 |
| Shipai Subdistrict | Tianhe | 0.20 | 0.26 | 0.31 |
| Shadong Subdistrict | Tianhe | 0.26 | 0.25 | 0.30 |
| Tianhe South Subdistrict | Tianhe | 0.15 | 0.19 | 0.24 |
| Linhe Subdistrict | Tianhe | 0.21 | 0.23 | 0.29 |
| Xinghua Subdistrict | Tianhe | 0.31 | 0.35 | 0.38 |
| Tangxia Subdistrict | Tianhe | 0.14 | 0.24 | 0.31 |
| Tianyuan Subdistrict | Tianhe | 0.15 | 0.25 | 0.32 |
| Liede Subdistrict | Tianhe | 0.14 | 0.22 | 0.29 |
| Xiancun Subdistrict | Tianhe | 0.33 | 0.40 | 0.43 |
| Yuangang Subdistrict | Tianhe | 0.27 | 0.32 | 0.36 |
| Huangcun Subdistrict | Tianhe | 0.31 | 0.39 | 0.40 |
| Changxing Subdistrict | Tianhe | 0.18 | 0.23 | 0.30 |
| Longdong Subdistrict | Tianhe | 0.20 | 0.31 | 0.38 |
| Fenghuang Subdistrict | Tianhe | 0.31 | 0.43 | 0.46 |
| Qianjin Subdistrict | Tianhe | 0.24 | 0.28 | 0.33 |
| Zhuji Subdistrict | Tianhe | 0.23 | 0.27 | 0.31 |
| Xintang Subdistrict | Tianhe | 0.31 | 0.38 | 0.41 |
| Sanyuanli Subdistrict | Baiyun | 0.37 | 0.31 | 0.32 |
| Songzhou Subdistrict | Baiyun | 0.43 | 0.34 | 0.33 |
| Jingtai Subdistrict | Baiyun | 0.39 | 0.30 | 0.32 |
| Tongde Subdistrict | Baiyun | 0.25 | 0.24 | 0.28 |
| Huangshi Subdistrict | Baiyun | 0.38 | 0.33 | 0.34 |
| Tangjing Subdistrict | Baiyun | 0.40 | 0.34 | 0.35 |
| Xinshi Subdistrict | Baiyun | 0.38 | 0.30 | 0.31 |
| Tonghe Subdistrict | Baiyun | 0.17 | 0.29 | 0.36 |
| Jingxi Subdistrict | Baiyun | 0.23 | 0.25 | 0.32 |
| Yongping Subdistrict | Baiyun | 0.39 | 0.36 | 0.37 |
| Jiahe Subdistrict | Baiyun | 0.24 | 0.21 | 0.26 |
| Junhe Subdistrict | Baiyun | 0.16 | 0.16 | 0.21 |
| Shijing Subdistrict | Baiyun | 0.39 | 0.37 | 0.35 |
| Jinsha Subdistrict | Baiyun | 0.31 | 0.24 | 0.26 |
| Yuncheng Subdistrict | Baiyun | 0.39 | 0.32 | 0.33 |
| Helong Subdistrict | Baiyun | 0.27 | 0.28 | 0.33 |
| Baiyunhu Subdistrict | Baiyun | 0.29 | 0.30 | 0.34 |
| Shimen Subdistrict | Baiyun | 0.38 | 0.37 | 0.35 |
| Longgui Subdistrict | Baiyun | 0.24 | 0.34 | 0.37 |
| Dayuan Subdistrict | Baiyun | 0.12 | 0.22 | 0.31 |
| Renhe Town | Baiyun | 0.20 | 0.32 | 0.37 |
| Taihe Town | Baiyun | 0.15 | 0.25 | 0.33 |
| Zhongluotan Town | Baiyun | 0.37 | 0.36 | 0.41 |
| Jianggao Town | Baiyun | 0.22 | 0.29 | 0.30 |
| Huangpu Subdistrict | Huangpu | 0.30 | 0.28 | 0.33 |
| Hongshan Subdistrict | Huangpu | 0.48 | 0.39 | 0.41 |
| Yuzhu Subdistrict | Huangpu | 0.42 | 0.33 | 0.35 |
| Dasha Subdistrict | Huangpu | 0.41 | 0.32 | 0.34 |
| Wenchong Subdistrict | Huangpu | 0.43 | 0.32 | 0.37 |
| Suidong Subdistrict | Huangpu | 0.42 | 0.28 | 0.29 |
| Nangang Subdistrict | Huangpu | 0.47 | 0.33 | 0.32 |
| Changzhou Subdistrict | Huangpu | 0.34 | 0.26 | 0.33 |
| Xiagang Subdistrict | Huangpu | 0.39 | 0.26 | 0.27 |
| Luogang Subdistrict | Huangpu | 0.40 | 0.44 | 0.41 |
| Yunpu Subdistrict | Huangpu | 0.40 | 0.43 | 0.44 |
| Lianhe Subdistrict | Huangpu | 0.20 | 0.34 | 0.39 |
| Yonghe Subdistrict | Huangpu | 0.48 | 0.42 | 0.31 |
| Changling Subdistrict | Huangpu | 0.39 | 0.43 | 0.43 |
| Jiufo Subdistrict | Huangpu | 0.70 | 0.28 | 0.27 |
| Longhu Subdistrict | Huangpu | 0.76 | 0.32 | 0.31 |
| Xinlong Town | Huangpu | 0.90 | 0.51 | 0.44 |
| Shiqiao Subdistrict | Panyu | 0.28 | 0.33 | 0.32 |
| Shatou Subdistrict | Panyu | 0.17 | 0.20 | 0.25 |
| Donghuan Subdistrict | Panyu | 0.29 | 0.34 | 0.34 |
| Qiaonan Subdistrict | Panyu | 0.23 | 0.22 | 0.20 |
| Xiaoguwei Subdistrict | Panyu | 0.22 | 0.20 | 0.26 |
| Dashi Subdistrict | Panyu | 0.11 | 0.18 | 0.24 |
| Luopu Subdistrict | Panyu | 0.20 | 0.24 | 0.28 |
| Shibei Subdistrict | Panyu | 0.13 | 0.17 | 0.22 |
| Zhongcun Subdistrict | Panyu | 0.16 | 0.23 | 0.28 |
| Dalong Subdistrict | Panyu | 0.22 | 0.31 | 0.29 |
| Nancun Town | Panyu | 0.22 | 0.27 | 0.31 |
| Xinzao Town | Panyu | 0.33 | 0.27 | 0.31 |
| Hualong Town | Panyu | 0.33 | 0.32 | 0.34 |
| Shilou Town | Panyu | 0.18 | 0.24 | 0.27 |
| Shawan Town | Panyu | 0.33 | 0.38 | 0.34 |
| Shiqi Town | Panyu | 0.31 | 0.38 | 0.34 |
| Xinhua Subdistrict | Huadu | 0.34 | 0.33 | 0.28 |
| Huacheng Subdistrict | Huadu | 0.36 | 0.33 | 0.29 |
| Xiuquan Subdistrict | Huadu | 0.36 | 0.29 | 0.24 |
| Xinya Subdistrict | Huadu | 0.31 | 0.33 | 0.29 |
| Timian Town | Huadu | 0.36 | 0.31 | 0.27 |
| Huashan Town | Huadu | 0.43 | 0.45 | 0.44 |
| Huadong Town | Huadu | 0.53 | 0.45 | 0.42 |
| Tanbu Town | Huadu | 0.24 | 0.21 | 0.22 |
| Chini Town | Huadu | 0.30 | 0.22 | 0.20 |
| Shiling Town | Huadu | 0.35 | 0.28 | 0.25 |
| Nansha Subdistrict | Nansha | 0.42 | 0.33 | 0.21 |
| Zhujiang Subdistrict | Nansha | 1.17 | 0.55 | 0.24 |
| Longxue Subdistrict | Nansha | 0.28 | 0.30 | 0.14 |
| Wanqingsha Town | Nansha | 1.15 | 0.53 | 0.23 |
| Hengli Town | Nansha | 0.60 | 0.37 | 0.21 |
| Huangge Town | Nansha | 0.53 | 0.35 | 0.26 |
| Dongchong Town | Nansha | 0.33 | 0.37 | 0.31 |
| Dagang Town | Nansha | 0.58 | 0.40 | 0.28 |
| Lanhe Town | Nansha | 0.46 | 0.27 | 0.24 |
| Jiekou Subdistrict | Conghua | 0.56 | 0.50 | 0.37 |
| Jiangpu Subdistrict | Conghua | 0.54 | 0.52 | 0.38 |
| Chengjiao Subdistrict | Conghua | 0.46 | 0.53 | 0.47 |
| Wenquan Town | Conghua | 0.64 | 0.51 | 0.34 |
| Liangkou Town | Conghua | 0.81 | 0.70 | 0.46 |
| Lvtian Town | Conghua | 1.08 | 0.80 | 0.44 |
| Taiping Town | Conghua | 0.60 | 0.47 | 0.46 |
| Aotou Town | Conghua | 0.61 | 0.45 | 0.28 |
| Licheng Subdistrict | Zengcheng | 0.56 | 0.49 | 0.34 |
| Zengjiang Subdistrict | Zengcheng | 0.50 | 0.44 | 0.33 |
| Zhucun Subdistrict | Zengcheng | 0.67 | 0.53 | 0.32 |
| Yongning Subdistrict | Zengcheng | 0.58 | 0.50 | 0.38 |
| Lihu Subdistrict | Zengcheng | 0.66 | 0.53 | 0.34 |
| Ningxi Subdistrict | Zengcheng | 0.66 | 0.53 | 0.34 |
| Xintang Town | Zengcheng | 0.33 | 0.33 | 0.28 |
| Shitan Town | Zengcheng | 0.38 | 0.38 | 0.27 |
| Zhongxin Town | Zengcheng | 0.98 | 0.51 | 0.42 |
| Zhengguo Town | Zengcheng | 0.07 | 0.22 | 0.23 |
| Paitan Town | Zengcheng | 0.12 | 0.29 | 0.30 |
| Xiaolou Town | Zengcheng | 0.38 | 0.51 | 0.42 |
| Xiancun Town | Zengcheng | 0.74 | 0.65 | 0.39 |

**Table S4** Spatial autocorrelation statistics under different thresholds.

| Thresholds | Moran’s I | z-score | *p*-value |
| --- | --- | --- | --- |
| $A_{i}$(d0=30 min) | 0.598 | 18.974 | <0.01 |
| $A_{i}$(d0=45 min) | 0.708 | 22.314 | <0.01 |
| $A_{i}$(d0=60 min) | 0.649 | 20.378 | <0.01 |

**Table S5** Sensitivity analyses of accessibility scores using different data of rabies exposure (during 2020-2022 *vs* during 2021-2022).

| Model | $A_{i}$ (95%CI) | | |
| --- | --- | --- | --- |
|  | Scenario 1  (d0=30 min) | Scenario 2  (d0=45 min) | Scenario 3  (d0=60 min) |
| Model I  (during 2020-2022) | 0.30(0.07~0.87) | 0.28(0.11~0.53) | 0.28(0.14~0.44) |
| Model II  (during 2021-2022) | 0.25(0.06~0.77) | 0.24(0.09~0.46) | 0.24(0.11~0.39) |
